# Whole-genome assembly of a novel invertebrate herpesvirus from the gastropod Babylonia areolata

**DOI:** 10.1099/mgen.0.001237

**Published:** 2024-04-24

**Authors:** Konstantin Divilov

**Affiliations:** 1 Department of Fisheries, Wildlife, and Conservation Sciences, Coastal Oregon Marine Experiment Station, Oregon State University, Hatfield Marine Science Center, Newport, Oregon 97365, USA

**Keywords:** genome assembly, HaHV-1, invertebrate, metagenomic, mollusc, OsHV-1

## Abstract

Molluscan herpesviruses cause disease in species of major importance to aquaculture and are the only known herpesviruses to infect invertebrates, which lack an adaptive immune system. Understanding the evolution of malacoherpesviruses in relation to their hosts will likely require comparative genomic studies on multiple phylogenetic scales. Currently, only two malacoherpesvirus species have genomes that have been fully assembled, which limits the ability to perform comparative genomic studies on this family of viruses. In the present study, we fully assemble a herpesvirus from Illumina and Nanopore sequence data that were previously used to assemble the genome of the gastropod *Babylonia areolata*. We tentatively assign this novel herpesvirus to the genus *Aurivirus* within the family *Malacoherpesviridae* based on a phylogenetic analysis of DNA polymerase. While structurally similar to other malacoherpesvirus genomes, a synteny analysis of the novel herpesvirus with another *Aurivirus* species indicates that genomic rearrangements might be an important process in the evolution of this genus. We anticipate that future complete assemblies of malacoherpesviruses will be a valuable resource in comparative herpesvirus research.

**Impact Statement**

Herpesviruses are an important group of viruses that negatively impact human and animal health. Molluscs are the hosts of disease-causing herpesviruses in the family *Malacoherpesviridae*, which is the only family of herpesviruses known to infect invertebrates. Despite their importance, only two *Malacoherpesviridae* species have fully assembled genomes. The lack of whole-genome information for this family of herpesviruses limits the ability to conduct comparative and evolutionary genomic studies. In this study, we fully assemble the genome of a novel *Malacoherpesviridae* species and highlight the role genomic rearrangements may have played in the evolution of this viral family.

## Data Summary

Nucleotide sequence data reported are available in the Third Party Annotation Section of the DDBJ/ENA/GenBank databases under the accession number TPA: BK064993.

## Introduction

Herpesviruses are dsDNA viruses that infect a wide range of animal hosts, establish lifelong latent infections, and often cause disease [1]. The order *Herpesvirales* is composed of three families: *Orthoherpesviridae* (*n*=118 species), *Alloherpesviridae* (*n*=13 species), and *Malacoherpesviridae* (*n*=2 species) [2]. Mammalian, avian, and reptilian herpesviruses belong to the *Orthoherpesviridae*; fish and amphibian herpesviruses belong to the *Alloherpesviridae*; and molluscan herpesviruses belong to the *Malacoherpesviridae* [3]. While herpesviruses are ubiquitous among vertebrates [4], they have not been widely found in molluscs. Currently, only two herpesviruses infecting molluscs, namely ostreid herpesvirus 1 (OsHV-1) and haliotid herpesvirus 1 (HaHV-1), have been identified [5,7] and their genomes fully assembled [8,9]. The primary hosts of OsHV-1 and HaHV-1 are the Pacific oyster (*Crassostrea gigas*) and abalone (*Haliotis* spp.), respectively, which are both among the most commonly grown molluscs in the world [10]. OsHV-1 and HaHV-1 were identified from animals suffering high mortality with the suspicion that the causal agent of the mortality might have been a virus, and this is an effective approach in identifying novel *Herpesvirales* spp. in molluscs. Recently, Rosani *et al*. [11] queried the NCBI SRA database taxonomy annotations to search for sequence data with malacoherpesvirus reads and produced four contig-level genome assemblies of novel herpesviruses (MalacoHV1-4), three of which were derived from water samples and the fourth from a blue mussel (*Mytilus edulis*) tissue sample. This study suggests that there are likely more malacoherpesviruses in existence than those presently identified; however, the incomplete contig-level genome assemblies of MalacoHV1-4 do not allow for a structure-based comparative analysis to be performed with existing herpesviruses.

## Results and Discussion

**Fig. 1.**
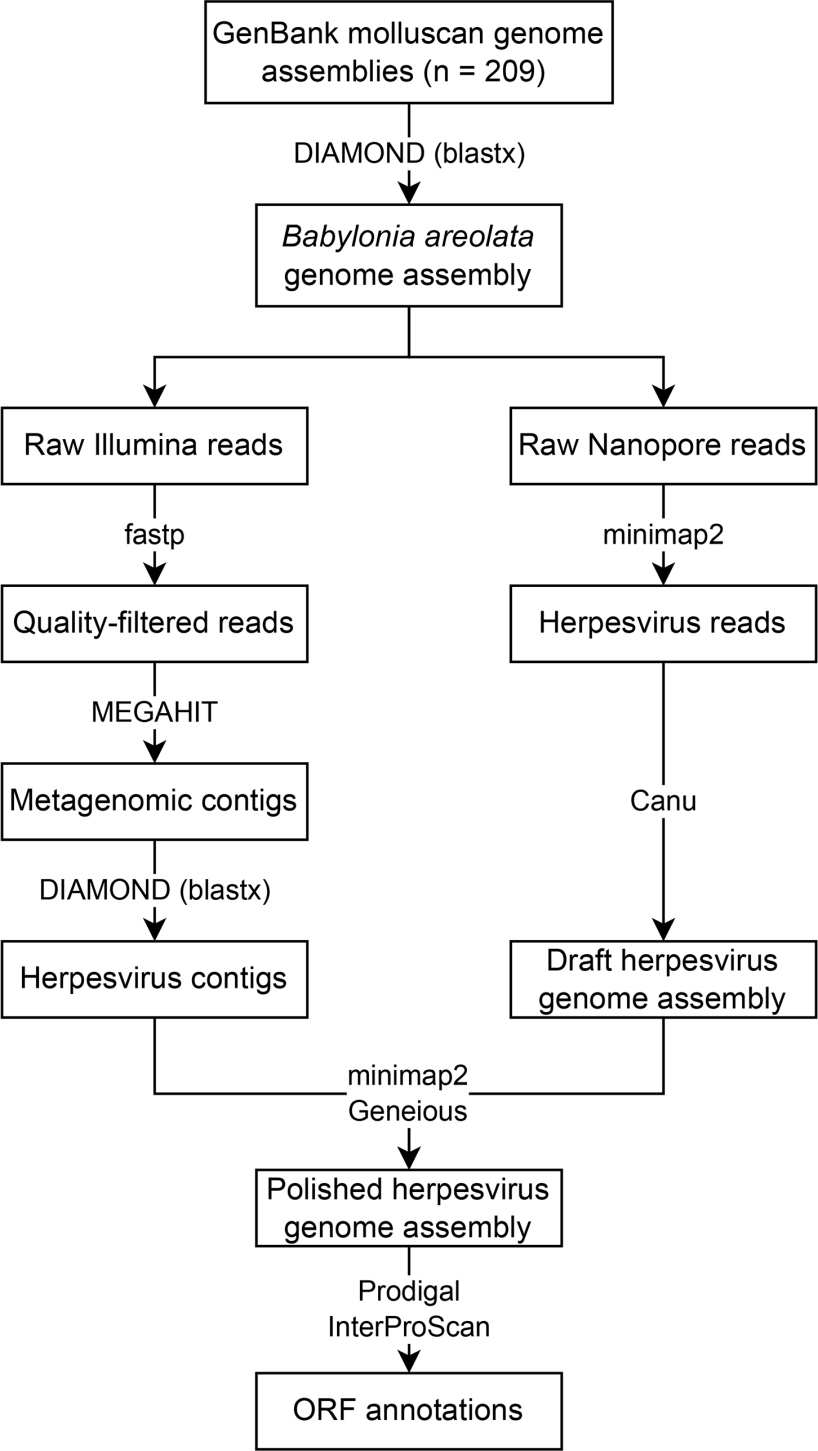
Bioinformatics workflow used for the identification of herpesvirus contigs from the *Babylonia areolata* genome assembly and the whole-genome assembly and annotation of this herpesvirus from raw Illumina and Nanopore reads.

In the present study, instead of querying the SRA database, we hypothesized that malacoherpesviruses likely have been co-assembled, at least partially, during the genome assembly of their molluscan hosts. Thus, we queried molluscan genome assemblies for the presence of malacoherpesviruses (Fig. 1). To do this, all NCBI GenBank genome assemblies in the phylum Mollusca (*n*=209 genomes) were downloaded using NCBI’s *datasets* tool on 15 June 2023. A translated blast (blastx) search of OsHV-1 ORFs (*n*=116) and HaHV-1 ORFs (*n*=112) was performed on the genome assemblies using DIAMOND v2.1.8 [12] with an E-value threshold of 1×10^−50^. The contig-level *Babylonia areolata* (GCA_011634625.1) genome assembly was the only assembly that contained a significant proportion of ORFs from either malacoherpesvirus, specifically 46 % of HaHV-1 ORFs were detected on 12 contigs of the genome assembly. *B. areolata* is a marine gastropod mollusc that is cultivated in Asia for seafood [13,14].

**Fig. 2.**
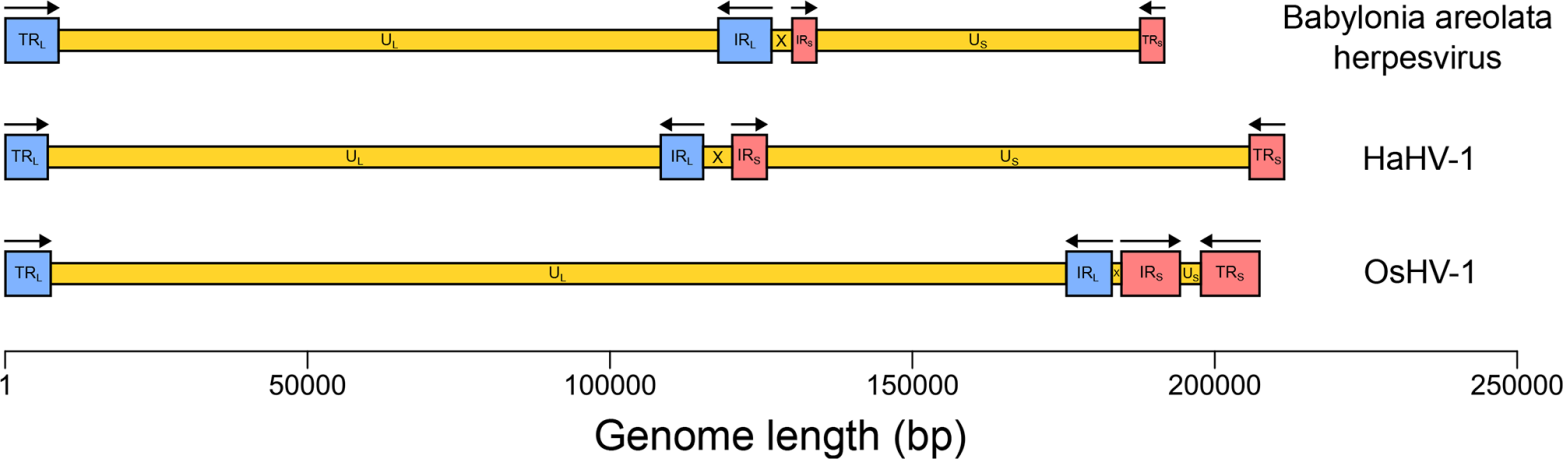
Genome structure of the *Babylonia areolata* herpesvirus (BaHV) assembled in the present study and the only other malacoherpesviruses with fully assembled genomes (HaHV-1 and OsHV-1).

In order to assemble the genome of the herpesvirus identified in the *B. areolata* genome assembly (Fig. 1), Illumina (2×150 bp) and Nanopore reads obtained from the *B. areolata* individual (BioSample SAMN12045496) used for this genome assembly were downloaded from NCBI (BioProject PRJNA548648). Using fastp v0.23.4 [15], Illumina reads were adapter trimmed, pruned using a 5′ and 3′ 4 bp sliding window (mean Phred quality score of 30), and filtered to keep reads with a minimum length of 100 bp and 90 % of bases at or above a Phred quality score of 30. The quality-filtered Illumina reads were then *de novo* assembled into contigs using MEGAHIT v1.2.9 [16]. A translated blast (blastx) search of HaHV-1 proteins was performed using DIAMOND on the assembled contigs to identify HaHV-1-like contigs. Afterward, Nanopore reads were mapped to these contigs using minimap2 v2.26 [17], and the mapped Nanopore reads were then assembled into two contigs that had a 6588 bp overlap using Canu v2.2 [18]. Scaffolding these two contigs using Geneious v2023.2 generated a draft assembly that had the same genome structure as HaHV-1 and OsHV-1, namely TR_L_-U_L_-IR_L_-X-IR_S_-U_S_-TR_S_ [8,9]. Inverted repeats were identified with the Repeat Finder plugin in Geneious. Five MEGAHIT-derived contigs were found to map to this draft genome using minimap2; one contig each spanned the entirety of the R_L_, U_L_, and U_S_ regions, while two contigs spanned the X and R_S_ regions. Geneious was then used to scaffold the mapped contigs into a high-quality genome assembly of the *Babylonia areolata* herpesvirus (BaHV). This genome assembly had a length of 1 91 698 bp, which was shorter than either HaHV-1 (2 11 518 bp) and OsHV-1 (2 07 439 bp); however, the GC content of this genome (44.1 %) was more similar to HaHV-1 (46.8 %) than OsHV-1 (38.7 %). Additionally, the lengths of the U_L_ and U_S_ regions in BaHV were more similar to those of HaHV-1 than those of OsHV-1, which has a very long U_L_ region and a very short U_S_ region (Fig. 2). Read coverage was assessed by mapping the Illumina and Nanopore reads using BWA v0.7.17 [19] and minimap2, respectively, to BaHV. Coverage was uniform across the genome except for the X region, where coverage was twice as high (Fig. S1), which has previously been observed by Burioli *et al*. [20] for OsHV-1. Capturing the whole genome of BaHV and other malacoherpesviruses in single Nanopore reads, as recently done for HSV-1 [21], will aid in understanding the configuration of the X region in the isomers of these viruses [8].

**Fig. 3.**
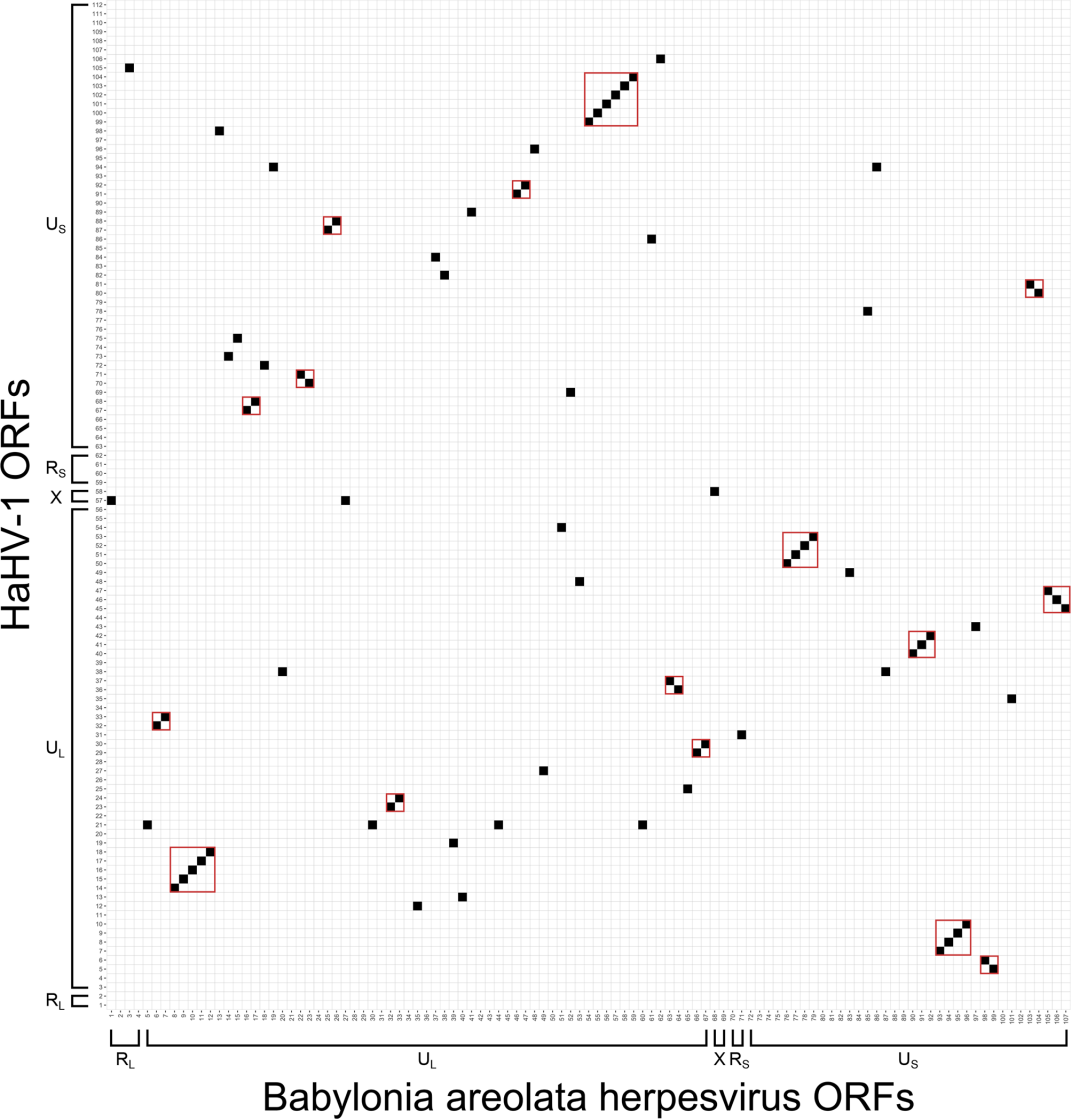
ORFs that are homologous between the *Babylonia areolata* herpesvirus (BaHV) assembled in the present study and HaHV-1. Syntenic regions are outlined by a red line.

ORFs in the BaHV genome were predicted using Prodigal v2.6.3 [22] and then tested for homology to RefSeq HaHV-1 and OsHV-1 ORFs using blastp [23] with an E-value threshold of 1×10^−5^. BaHV showed more synteny with HaHV-1 (*n*=16 regions) than OsHV-1 (*n*=8 regions) (Figs3  4). Among the 107 predicted BaHV ORFs, 75 and 45 % of them showed homology to HaHV-1 and OsHV-1 ORFs, respectively. Using InterProScan v5.64–96.0 [24] to annotate BaHV, we found that only zing finger (ZNF) annotations had >2 ORF annotations in each malacoherpesvirus, with both BaHV and HaHV-1 having 3 ZNF ORFs and OsHV-1 having 10 ZNF ORFs. A phylogenetic tree of DNA polymerase proteins from BaHV, *Malacoherpesviridae* species, and *Alloherpesviridae* species was constructed using muscle v5.1 [25] and MrBayes v3.2 [26], and indicated that BaHV was most closely related to HaHV-1 (Fig. 5). Additionally, with the branch lengths of *Alloherpesviridae* species as a reference, BaHV can be inferred to represent a novel species within the genus *Aurivirus*.

**Fig. 4.**
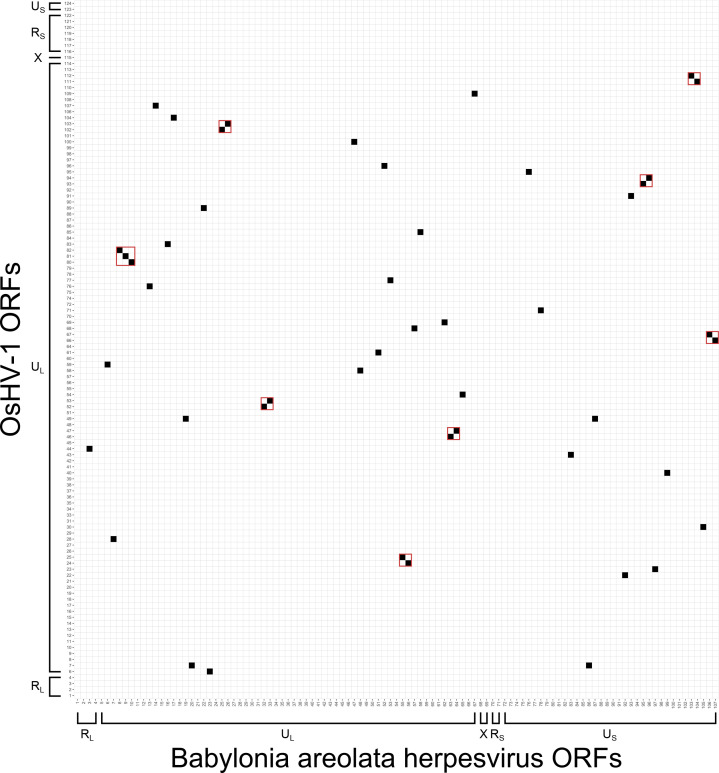
ORFs that are homologous between the *Babylonia areolata* herpesvirus (BaHV) assembled in the present study and OsHV-1. Syntenic regions are outlined by a red line.

**Fig. 5.**
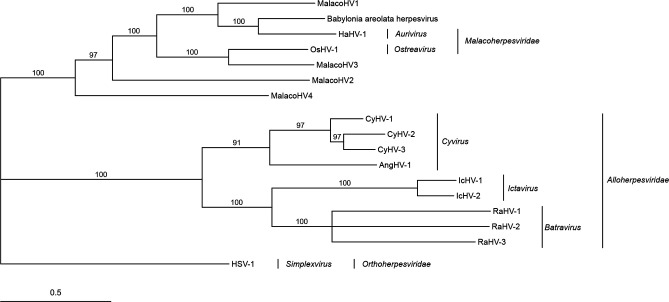
Phylogenetic tree built using DNA polymerases from the *Babylonia areolata* herpesvirus (BaHV) assembled in the present study, RefSeq *Alloherpesviridae* and *Malacoherpesviridae* species, and four malacoherpesviruses assembled at the contig level by Rosani *et al*. [11] (MalacoHV1-4), with HSV-1 as the outgroup. A general time-reversible model was used with four Markov chains of 1 00 000 samples each. The units of branch length are expected changes per site.

The genome of the herpesvirus assembled from *B. areolata* represents the third fully assembled genome of a malacoherpesvirus species. Despite having the same genome structure as other malacoherpesviruses, substantial differences in ORF content, synteny, and region lengths were present. No homologous ORFs were found between the R_L_ and R_S_ regions in BaHV and HaHV-1, as well as OsHV-1, which suggests that ORFs in these regions are unlikely to have conserved function. One of two ORFs in the X region of BaHV was homologous to one of two ORFs in the X region of HaHV-1, suggesting a conserved functional role for the X region; however, this ORF could not be functionally annotated. The U_L_ and U_S_ lengths of BaHV were more similar to HaHV-1 than OsHV-1, but unexpectedly, the U_L_ regions of BaHV and HaHV-1 were not more syntenic compared to the U_L_ and U_S_ regions of both genomes, and the U_S_ regions of BaHV and HaHV-1 were less syntenic compared to the U_L_ and U_S_ regions of both genomes. The poor synteny between the U_S_ regions of BaHV and HaHV-1 may be due to the U_S_ region of these viruses being more likely to undergo genomic rearrangement than the U_L_ region; however, it could also be due to stochasticity in the evolution of both viruses from their last common ancestor. Obtaining more whole-genome assemblies of novel malacoherpesviruses would be one way to better understand the historical evolutionary processes that gave rise to the present suite of viral genomes.

The discovery of malacoherpesviruses in the current study and by Rosani *et al*. [11] from animals (*B. areolata* and *M. edulis*) that were not identified as presenting disease symptoms suggests that either they were experiencing asymptomatic primary infections or asymptomatic reactivation of persistent/latent infections [27]. Given that genome assembly projects choose healthy, unstressed individuals for sequencing, the approach used in this study to find herpesviruses likely has a large false-negative rate. Attempting to reactivate any potential persistent/latent infections prior to sample collection destined for whole-genome sequencing would likely increase sequencing coverage of any present herpesviruses and make whole-genome assembly more feasible. Additionally, pan-malacoherpesvirus consensus primers [28,29] could be developed and used as a test of presence/absence before sequencing. The results of this study indicate that a thorough search for malacoherpesviruses will likely uncover more novel herpesviruses, which will help drive comparative genomic studies to better understand this family of herpesviruses that impact on animal health.

## supplementary material

10.1099/mgen.0.001237 Fig. S1.

## Abbreviations

BaHV: *Babylonia areolata* herpesvirus
HaHV-1: haliotid herpesvirus 1
HSV-1: herpes simplex virus 1
OsHV-1: ostreid herpesvirus 1
ZNF: Zinc finger
