# Supplementary figures and images for: Whole-genome assembly of a novel invertebrate herpesvirus from the gastropod Babylonia areolata
Source: Microb Genom. 2024 Apr 24;10(4):001237. doi: 10.1099/mgen.0.001237 (PMC11092209; doi:10.1099/mgen.0.001237)

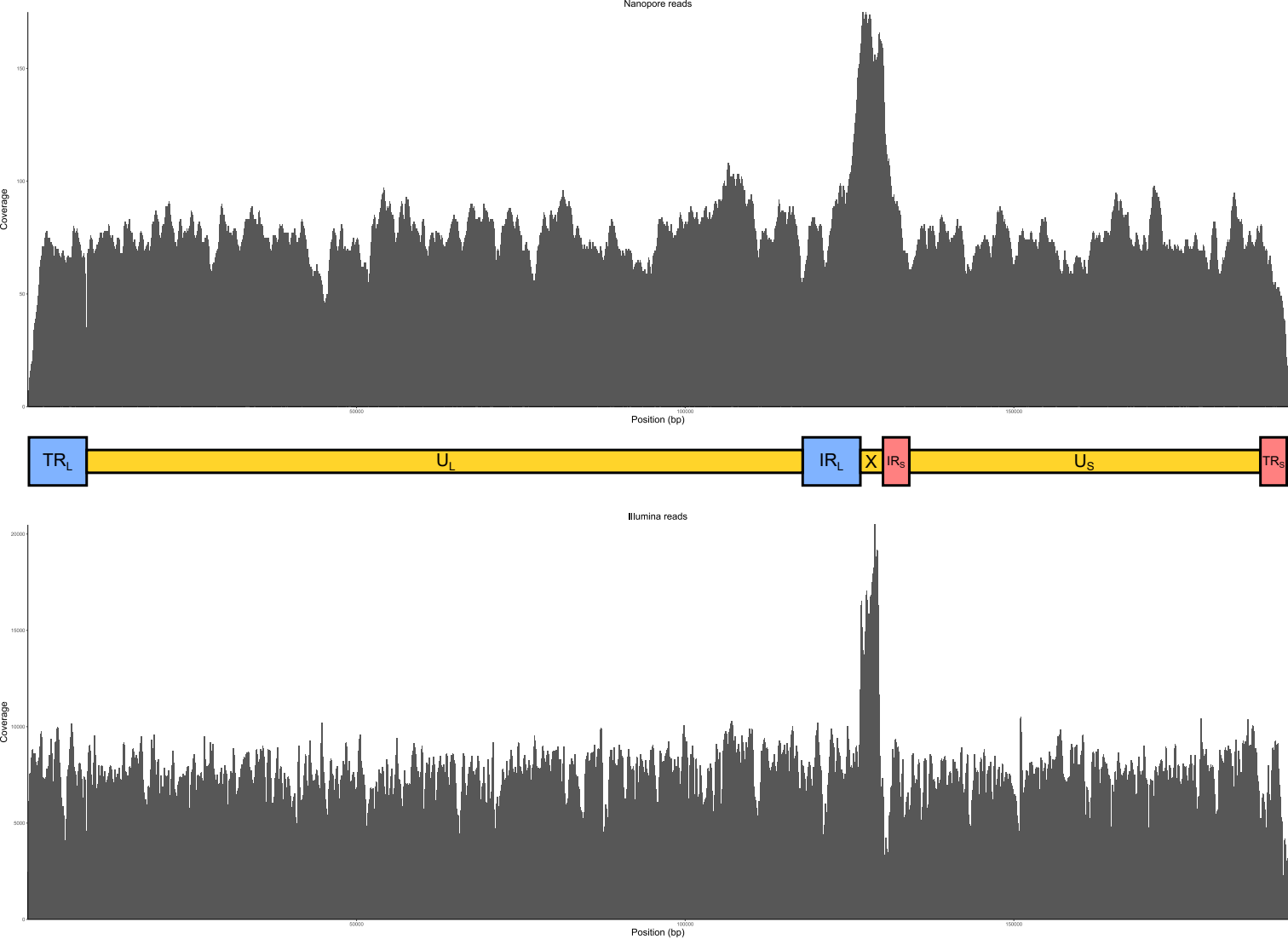

Fig. S1. Nanopore and Illumina read coverage of the Babylonia areolata herpesvirus.

Supplement: Fig. S1. [file mgen-10-01237-s001.pdf]
